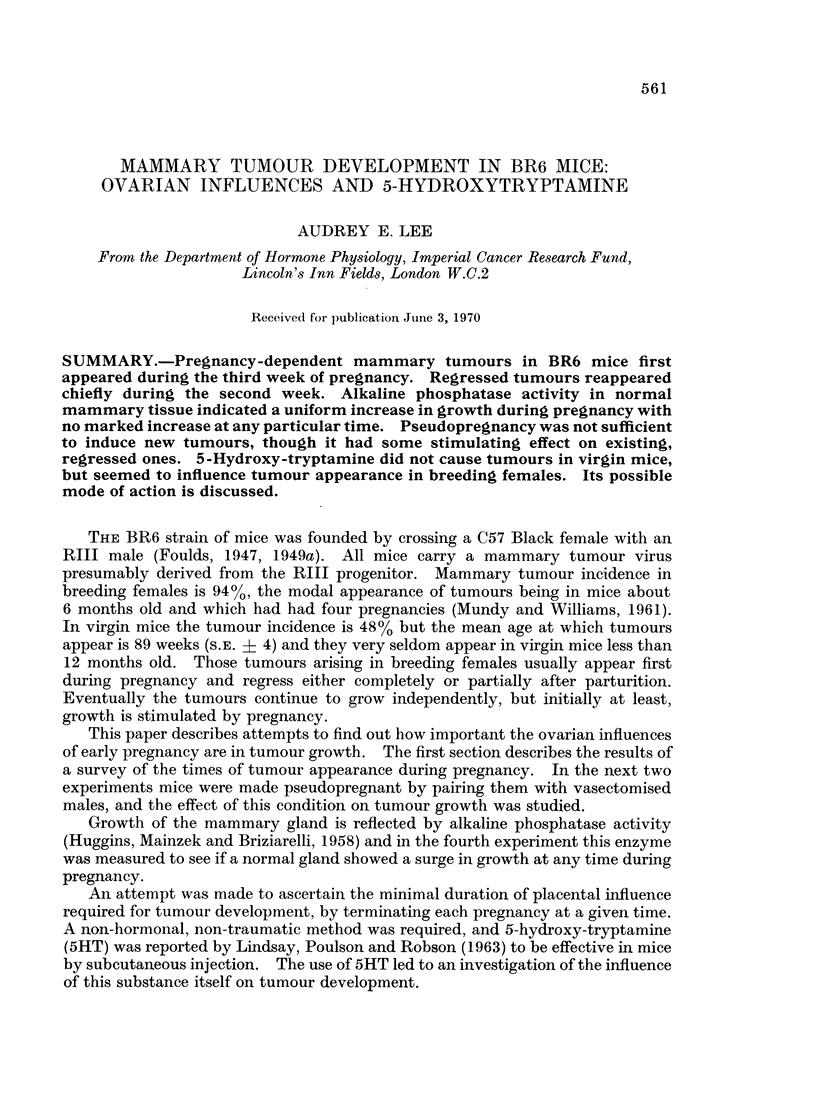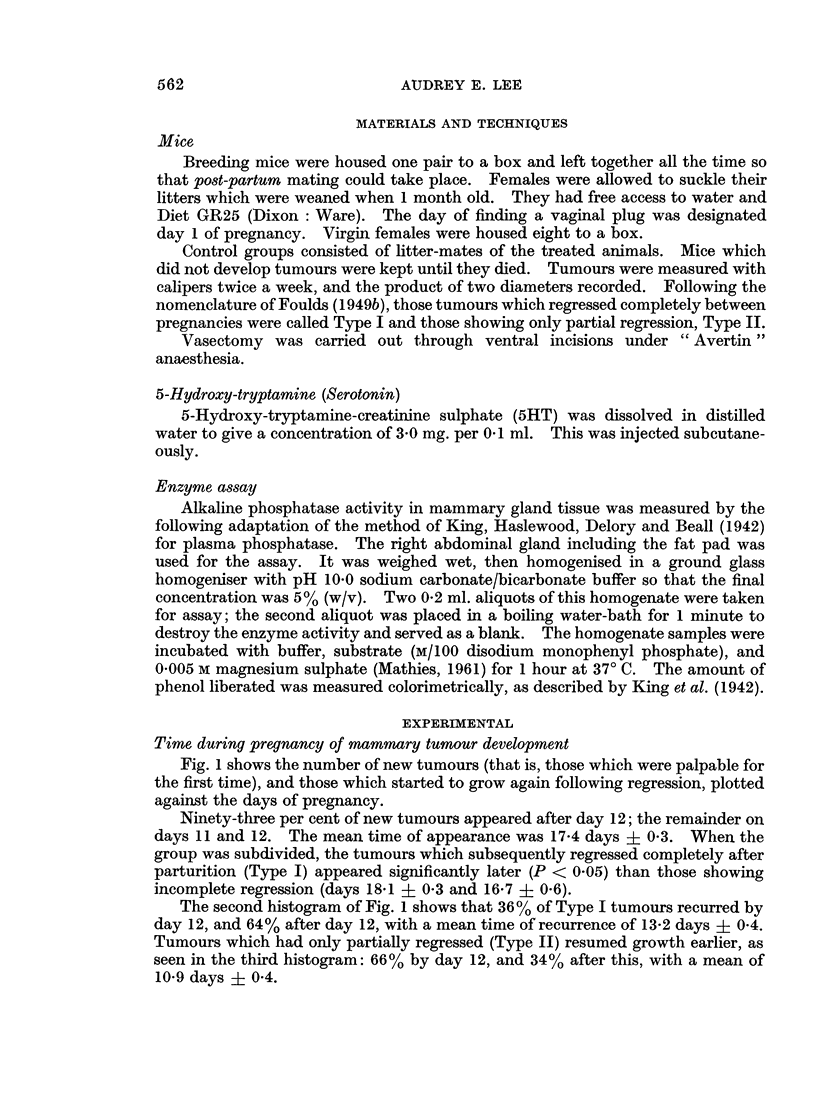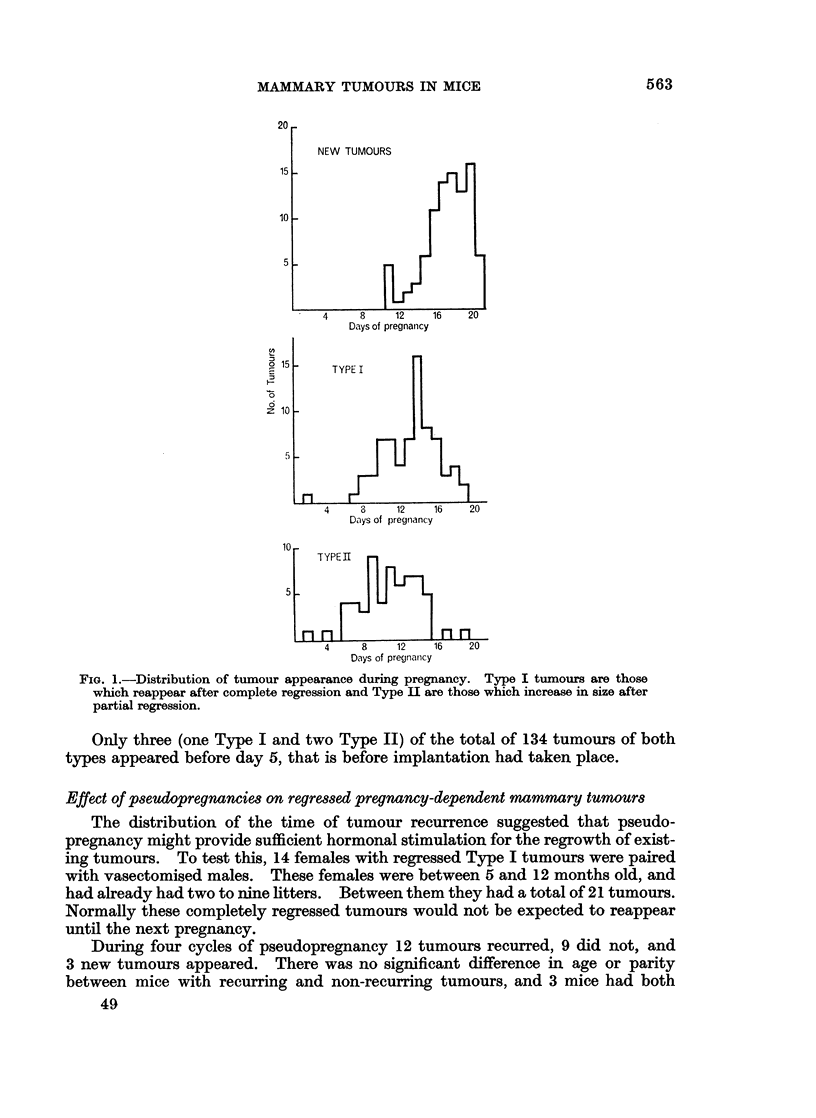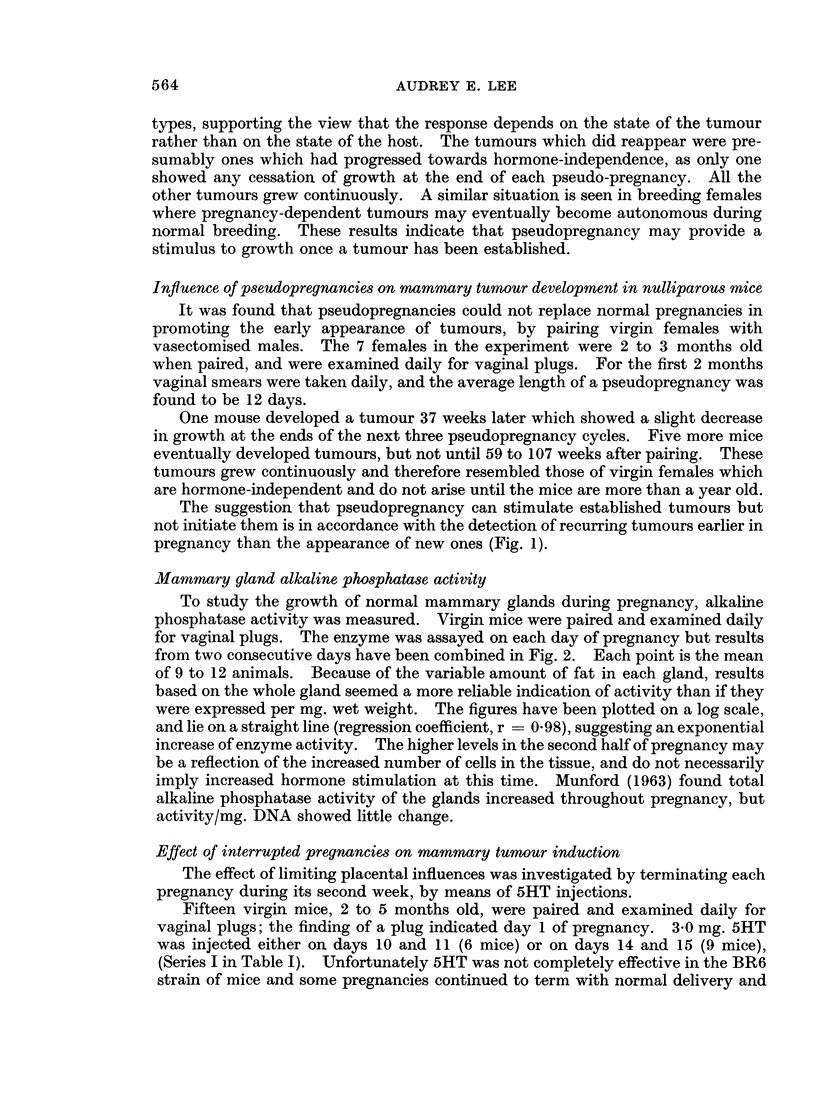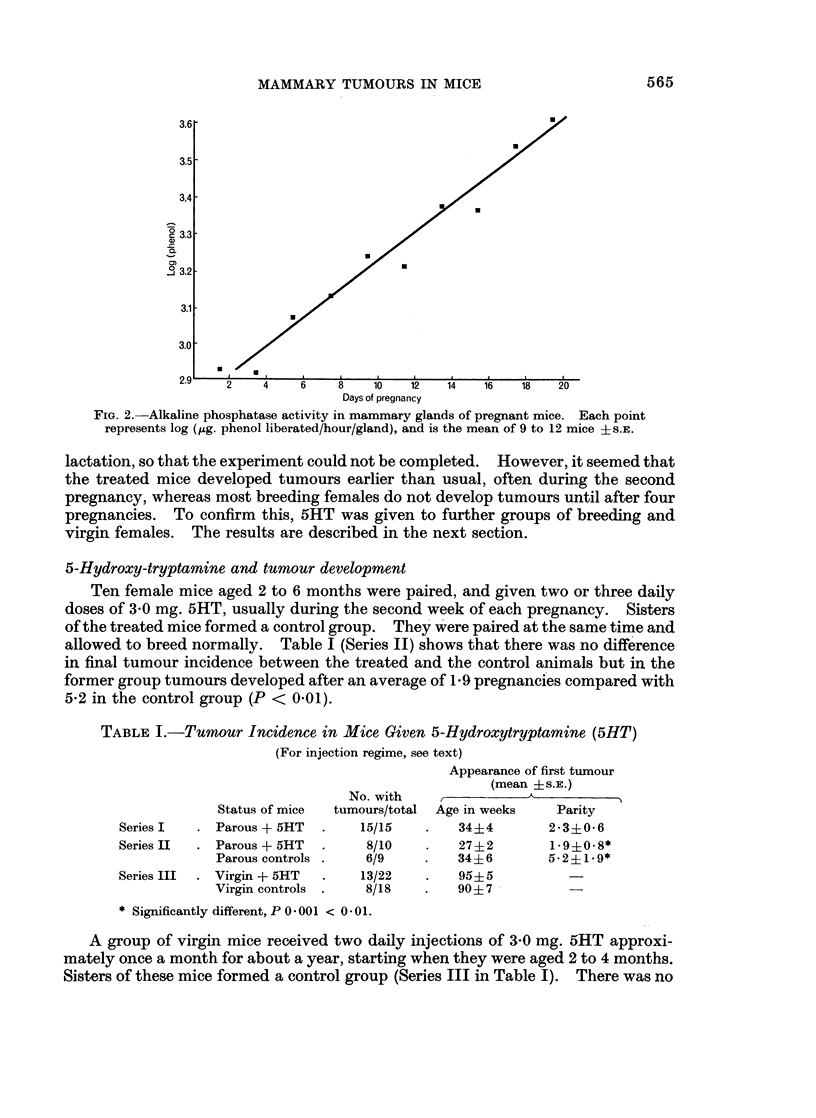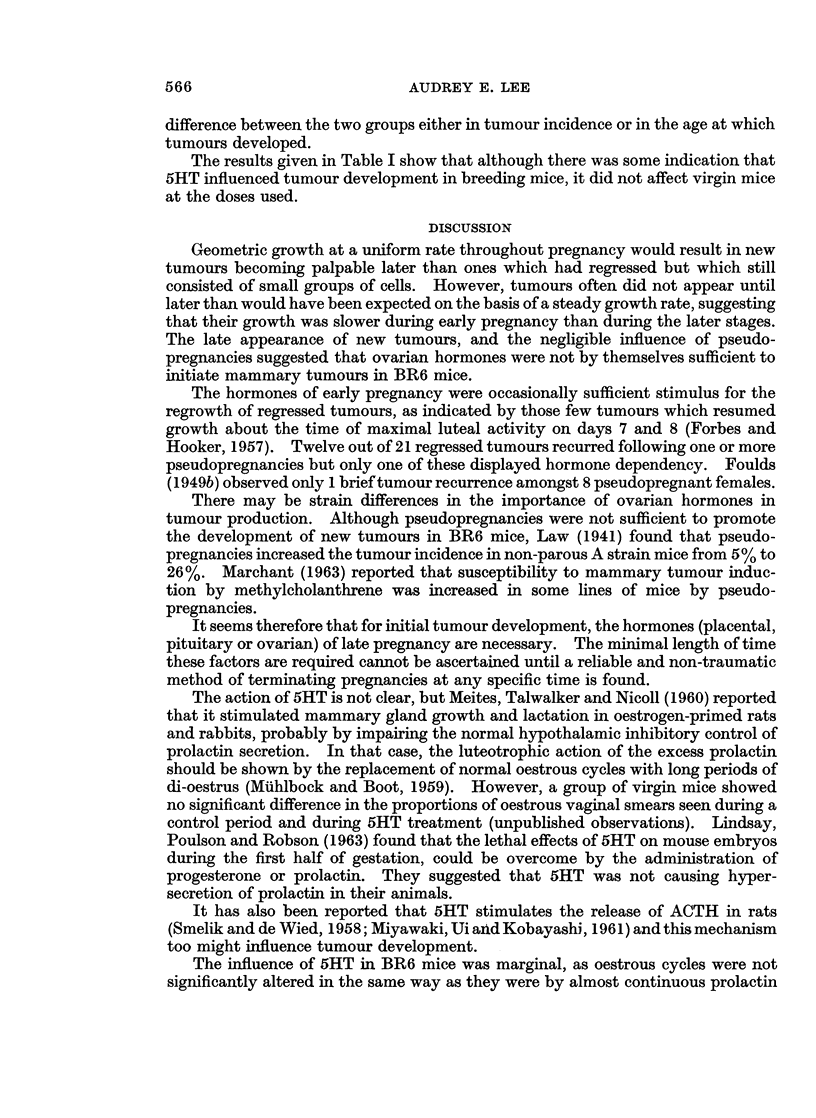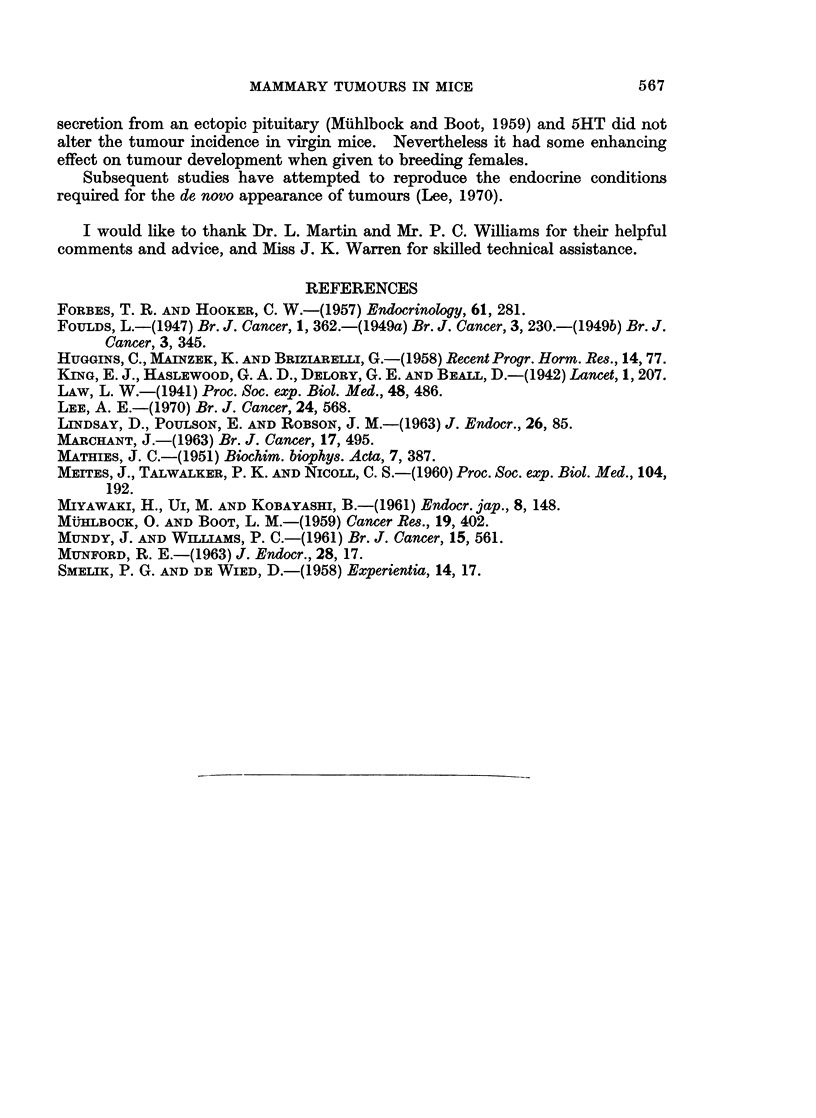# Mammary Tumour Development in BR6 Mice: Ovarian Influences and 5-Hydroxytryptamine

**DOI:** 10.1038/bjc.1970.68

**Published:** 1970-09

**Authors:** Audrey E. Lee

## Abstract

Pregnancy-dependent mammary tumours in BR6 mice first appeared during the third week of pregnancy. Regressed tumours reappeared chiefly during the second week. Alkaline Phosphatase activity in normal mammary tissue indicated a uniform increase in growth during pregnancy with no marked increase at any particular time. Pseudopregnancy was not sufficient to induce new tumours, though it had some stimulating effect on existing, regressed ones. 5-Hydroxy-tryptamine did not cause tumours in virgin mice, but seemed to influence tumour appearance in breeding females. Its possible mode of action is discussed.


### MAMMARY TUMOUR DEVELOPMENT IN BR6 MICE: OVARIAN INFLUENCES AND 5-HYDROXYTRYPTAMINE

AUDREY E. LEE

*From the Department of Hormone Physiology, Imperial Cancer Research Fund, Lincoln's Inn Fields, London W.C.2*

Received for publication June 3, 1970

**SUMMARY.—Pregnancy-dependent mammary tumours in BR6 mice first appeared during the third week of pregnancy. Regressed tumours reappeared chiefly during the second week. Alkaline phosphatase activity in normal mammary tissue indicated a uniform increase in growth during pregnancy with no marked increase at any particular time. Pseudopregnancy was not sufficient to induce new tumours, though it had some stimulating effect on existing, regressed ones. 5-Hydroxy-tryptamine did not cause tumours in virgin mice, but seemed to influence tumour appearance in breeding females. Its possible mode of action is discussed.**

THE BR6 strain of mice was founded by crossing a C57 Black female with an RIII male (Foulds, 1947, 1949*a*). All mice carry a mammary tumour virus presumably derived from the RIII progenitor. Mammary tumour incidence in breeding females is 94%, the modal appearance of tumours being in mice about 6 months old and which had had four pregnancies (Mundy and Williams, 1961). In virgin mice the tumour incidence is 48% but the mean age at which tumours appear is 89 weeks (S.E. $\pm$ 4) and they very seldom appear in virgin mice less than 12 months old. Those tumours arising in breeding females usually appear first during pregnancy and regress either completely or partially after parturition. Eventually the tumours continue to grow independently, but initially at least, growth is stimulated by pregnancy.

This paper describes attempts to find out how important the ovarian influences of early pregnancy are in tumour growth. The first section describes the results of a survey of the times of tumour appearance during pregnancy. In the next two experiments mice were made pseudopregnant by pairing them with vasectomised males, and the effect of this condition on tumour growth was studied.

Growth of the mammary gland is reflected by alkaline phosphatase activity (Huggins, Mainzek and Briziarelli, 1958) and in the fourth experiment this enzyme was measured to see if a normal gland showed a surge in growth at any time during pregnancy.

An attempt was made to ascertain the minimal duration of placental influence required for tumour development, by terminating each pregnancy at a given time. A non-hormonal, non-traumatic method was required, and 5-hydroxy-tryptamine (5HT) was reported by Lindsay, Poulson and Robson (1963) to be effective in mice by subcutaneous injection. The use of 5HT led to an investigation of the influence of this substance itself on tumour development.

### MATERIALS AND TECHNIQUES

### *Mice*

Breeding mice were housed one pair to a box and left together all the time so that *post-partum* mating could take place. Females were allowed to suckle their litters which were weaned when 1 month old. They had free access to water and Diet GR25 (Dixon : Ware). The day of finding a vaginal plug was designated day 1 of pregnancy. Virgin females were housed eight to a box.

Control groups consisted of litter-mates of the treated animals. Mice which did not develop tumours were kept until they died. Tumours were measured with calipers twice a week, and the product of two diameters recorded. Following the nomenclature of Foulds (1949*b*), those tumours which regressed completely between pregnancies were called Type I and those showing only partial regression, Type II.

Vasectomy was carried out through ventral incisions under "Avertin" anaesthesia.

### 5-*Hydroxy-tryptamine* (*Serotonin*)

5-Hydroxy-tryptamine-creatinine sulphate (5HT) was dissolved in distilled water to give a concentration of 3·0 mg. per 0·1 ml. This was injected subcutaneously.

### *Enzyme assay*

Alkaline phosphatase activity in mammary gland tissue was measured by the following adaptation of the method of King, Haslewood, Delory and Beall (1942) for plasma phosphatase. The right abdominal gland including the fat pad was used for the assay. It was weighed wet, then homogenised in a ground glass homogeniser with pH 10·0 sodium carbonate/bicarbonate buffer so that the final concentration was 5% (w/v). Two 0·2 ml. aliquots of this homogenate were taken for assay; the second aliquot was placed in a boiling water-bath for 1 minute to destroy the enzyme activity and served as a blank. The homogenate samples were incubated with buffer, substrate (M/100 disodium monophenyl phosphate), and 0·005 M magnesium sulphate (Mathies, 1961) for 1 hour at 37° C. The amount of phenol liberated was measured colorimetrically, as described by King *et al.* (1942).

### EXPERIMENTAL

### *Time during pregnancy of mammary tumour development*

Fig. 1 shows the number of new tumours (that is, those which were palpable for the first time), and those which started to grow again following regression, plotted against the days of pregnancy.

Ninety-three per cent of new tumours appeared after day 12; the remainder on days 11 and 12. The mean time of appearance was 17·4 days $\pm$ 0·3. When the group was subdivided, the tumours which subsequently regressed completely after parturition (Type I) appeared significantly later ($P < 0{\cdot}05$) than those showing incomplete regression (days 18·1 $\pm$ 0·3 and 16·7 $\pm$ 0·6).

The second histogram of Fig. 1 shows that 36% of Type I tumours recurred by day 12, and 64% after day 12, with a mean time of recurrence of 13·2 days $\pm$ 0·4. Tumours which had only partially regressed (Type II) resumed growth earlier, as seen in the third histogram: 66% by day 12, and 34% after this, with a mean of 10·9 days $\pm$ 0·4.

FIG. 1.—Distribution of tumour appearance during pregnancy. Type I tumours are those which reappear after complete regression and Type II are those which increase in size after partial regression.

Only three (one Type I and two Type II) of the total of 134 tumours of both types appeared before day 5, that is before implantation had taken place.

*Effect of pseudopregnancies on regressed pregnancy-dependent mammary tumours*

The distribution of the time of tumour recurrence suggested that pseudopregnancy might provide sufficient hormonal stimulation for the regrowth of existing tumours. To test this, 14 females with regressed Type I tumours were paired with vasectomised males. These females were between 5 and 12 months old, and had already had two to nine litters. Between them they had a total of 21 tumours. Normally these completely regressed tumours would not be expected to reappear until the next pregnancy.

During four cycles of pseudopregnancy 12 tumours recurred, 9 did not, and 3 new tumours appeared. There was no significant difference in age or parity between mice with recurring and non-recurring tumours, and 3 mice had both types, supporting the view that the response depends on the state of the tumour rather than on the state of the host. The tumours which did reappear were presumably ones which had progressed towards hormone-independence, as only one showed any cessation of growth at the end of each pseudo-pregnancy. All the other tumours grew continuously. A similar situation is seen in breeding females where pregnancy-dependent tumours may eventually become autonomous during normal breeding. These results indicate that pseudopregnancy may provide a stimulus to growth once a tumour has been established.

*Influence of pseudopregnancies on mammary tumour development in nulliparous mice*

It was found that pseudopregnancies could not replace normal pregnancies in promoting the early appearance of tumours, by pairing virgin females with vasectomised males. The 7 females in the experiment were 2 to 3 months old when paired, and were examined daily for vaginal plugs. For the first 2 months vaginal smears were taken daily, and the average length of a pseudopregnancy was found to be 12 days.

One mouse developed a tumour 37 weeks later which showed a slight decrease in growth at the ends of the next three pseudopregnancy cycles. Five more mice eventually developed tumours, but not until 59 to 107 weeks after pairing. These tumours grew continuously and therefore resembled those of virgin females which are hormone-independent and do not arise until the mice are more than a year old.

The suggestion that pseudopregnancy can stimulate established tumours but not initiate them is in accordance with the detection of recurring tumours earlier in pregnancy than the appearance of new ones (Fig. 1).

*Mammary gland alkaline phosphatase activity*

To study the growth of normal mammary glands during pregnancy, alkaline phosphatase activity was measured. Virgin mice were paired and examined daily for vaginal plugs. The enzyme was assayed on each day of pregnancy but results from two consecutive days have been combined in Fig. 2. Each point is the mean of 9 to 12 animals. Because of the variable amount of fat in each gland, results based on the whole gland seemed a more reliable indication of activity than if they were expressed per mg. wet weight. The figures have been plotted on a log scale, and lie on a straight line (regression coefficient, $r = 0{\cdot}98$), suggesting an exponential increase of enzyme activity. The higher levels in the second half of pregnancy may be a reflection of the increased number of cells in the tissue, and do not necessarily imply increased hormone stimulation at this time. Munford (1963) found total alkaline phosphatase activity of the glands increased throughout pregnancy, but activity/mg. DNA showed little change.

*Effect of interrupted pregnancies on mammary tumour induction*

The effect of limiting placental influences was investigated by terminating each pregnancy during its second week, by means of 5HT injections.

Fifteen virgin mice, 2 to 5 months old, were paired and examined daily for vaginal plugs; the finding of a plug indicated day 1 of pregnancy. 3·0 mg. 5HT was injected either on days 10 and 11 (6 mice) or on days 14 and 15 (9 mice), (Series I in Table I). Unfortunately 5HT was not completely effective in the BR6 strain of mice and some pregnancies continued to term with normal delivery and

FIG. 2.—Alkaline phosphatase activity in mammary glands of pregnant mice. Each point represents log ($\mu$g. phenol liberated/hour/gland), and is the mean of 9 to 12 mice ±S.E.

lactation, so that the experiment could not be completed. However, it seemed that the treated mice developed tumours earlier than usual, often during the second pregnancy, whereas most breeding females do not develop tumours until after four pregnancies. To confirm this, 5HT was given to further groups of breeding and virgin females. The results are described in the next section.

*5-Hydroxy-tryptamine and tumour development*

Ten female mice aged 2 to 6 months were paired, and given two or three daily doses of 3·0 mg. 5HT, usually during the second week of each pregnancy. Sisters of the treated mice formed a control group. They were paired at the same time and allowed to breed normally. Table I (Series II) shows that there was no difference in final tumour incidence between the treated and the control animals but in the former group tumours developed after an average of 1·9 pregnancies compared with 5·2 in the control group ($P < 0{\cdot}01$).

TABLE I.—*Tumour Incidence in Mice Given 5-Hydroxytryptamine (5HT)*

(For injection regime, see text)

| | Status of mice | No. with tumours/total | Appearance of first tumour (mean ±S.E.) Age in weeks | Parity |
|---|---|---|---|---|
| Series I | Parous + 5HT | 15/15 | 34±4 | 2·3±0·6 |
| Series II | Parous + 5HT | 8/10 | 27±2 | 1·9±0·8* |
| | Parous controls | 6/9 | 34±6 | 5·2±1·9* |
| Series III | Virgin + 5HT | 13/22 | 95±5 | — |
| | Virgin controls | 8/18 | 90±7 | — |

* Significantly different, $P$ 0·001 < 0·01.

A group of virgin mice received two daily injections of 3·0 mg. 5HT approximately once a month for about a year, starting when they were aged 2 to 4 months. Sisters of these mice formed a control group (Series III in Table I). There was no difference between the two groups either in tumour incidence or in the age at which tumours developed.

The results given in Table I show that although there was some indication that 5HT influenced tumour development in breeding mice, it did not affect virgin mice at the doses used.

### DISCUSSION

Geometric growth at a uniform rate throughout pregnancy would result in new tumours becoming palpable later than ones which had regressed but which still consisted of small groups of cells. However, tumours often did not appear until later than would have been expected on the basis of a steady growth rate, suggesting that their growth was slower during early pregnancy than during the later stages. The late appearance of new tumours, and the negligible influence of pseudopregnancies suggested that ovarian hormones were not by themselves sufficient to initiate mammary tumours in BR6 mice.

The hormones of early pregnancy were occasionally sufficient stimulus for the regrowth of regressed tumours, as indicated by those few tumours which resumed growth about the time of maximal luteal activity on days 7 and 8 (Forbes and Hooker, 1957). Twelve out of 21 regressed tumours recurred following one or more pseudopregnancies but only one of these displayed hormone dependency. Foulds (1949*b*) observed only 1 brief tumour recurrence amongst 8 pseudopregnant females.

There may be strain differences in the importance of ovarian hormones in tumour production. Although pseudopregnancies were not sufficient to promote the development of new tumours in BR6 mice, Law (1941) found that pseudopregnancies increased the tumour incidence in non-parous A strain mice from 5% to 26%. Marchant (1963) reported that susceptibility to mammary tumour induction by methylcholanthrene was increased in some lines of mice by pseudopregnancies.

It seems therefore that for initial tumour development, the hormones (placental, pituitary or ovarian) of late pregnancy are necessary. The minimal length of time these factors are required cannot be ascertained until a reliable and non-traumatic method of terminating pregnancies at any specific time is found.

The action of 5HT is not clear, but Meites, Talwalker and Nicoll (1960) reported that it stimulated mammary gland growth and lactation in oestrogen-primed rats and rabbits, probably by impairing the normal hypothalamic inhibitory control of prolactin secretion. In that case, the luteotrophic action of the excess prolactin should be shown by the replacement of normal oestrous cycles with long periods of di-oestrus (Mühlbock and Boot, 1959). However, a group of virgin mice showed no significant difference in the proportions of oestrous vaginal smears seen during a control period and during 5HT treatment (unpublished observations). Lindsay, Poulson and Robson (1963) found that the lethal effects of 5HT on mouse embryos during the first half of gestation, could be overcome by the administration of progesterone or prolactin. They suggested that 5HT was not causing hypersecretion of prolactin in their animals.

It has also been reported that 5HT stimulates the release of ACTH in rats (Smelik and de Wied, 1958; Miyawaki, Ui and Kobayashi, 1961) and this mechanism too might influence tumour development.

The influence of 5HT in BR6 mice was marginal, as oestrous cycles were not significantly altered in the same way as they were by almost continuous prolactin secretion from an ectopic pituitary (Mühlbock and Boot, 1959) and 5HT did not alter the tumour incidence in virgin mice. Nevertheless it had some enhancing effect on tumour development when given to breeding females.

Subsequent studies have attempted to reproduce the endocrine conditions required for the *de novo* appearance of tumours (Lee, 1970).

I would like to thank Dr. L. Martin and Mr. P. C. Williams for their helpful comments and advice, and Miss J. K. Warren for skilled technical assistance.

### REFERENCES

FORBES, T. R. AND HOOKER, C. W.—(1957) *Endocrinology*, **61**, 281.
FOULDS, L.—(1947) *Br. J. Cancer*, **1**, 362.—(1949*a*) *Br. J. Cancer*, **3**, 230.—(1949*b*) *Br. J. Cancer*, **3**, 345.
HUGGINS, C., MAINZEK, K. AND BRIZIARELLI, G.—(1958) *Recent Progr. Horm. Res.*, **14**, 77.
KING, E. J., HASLEWOOD, G. A. D., DELORY, G. E. AND BEALL, D.—(1942) *Lancet*, **1**, 207.
LAW, L. W.—(1941) *Proc. Soc. exp. Biol. Med.*, **48**, 486.
LEE, A. E.—(1970) *Br. J. Cancer*, **24**, 568.
LINDSAY, D., POULSON, E. AND ROBSON, J. M.—(1963) *J. Endocr.*, **26**, 85.
MARCHANT, J.—(1963) *Br. J. Cancer*, **17**, 495.
MATHIES, J. C.—(1951) *Biochim. biophys. Acta*, **7**, 387.
MEITES, J., TALWALKER, P. K. AND NICOLL, C. S.—(1960) *Proc. Soc. exp. Biol. Med.*, **104**, 192.
MIYAWAKI, H., UI, M. AND KOBAYASHI, B.—(1961) *Endocr. jap.*, **8**, 148.
MÜHLBOCK, O. AND BOOT, L. M.—(1959) *Cancer Res.*, **19**, 402.
MUNDY, J. AND WILLIAMS, P. C.—(1961) *Br. J. Cancer*, **15**, 561.
MUNFORD, R. E.—(1963) *J. Endocr.*, **28**, 17.
SMELIK, P. G. AND DE WIED, D.—(1958) *Experientia*, **14**, 17.